# Macroevolution of arboreality in salamanders

**DOI:** 10.1002/ece3.5267

**Published:** 2019-05-26

**Authors:** Erica K. Baken, Dean C. Adams

**Affiliations:** ^1^ Department of Ecology, Evolution, and Organismal Biology Iowa State University Ames Iowa

**Keywords:** amphibian, macroevolution, microhabitat, phylogenetic comparative methods

## Abstract

Evolutionary theory predicts that selection in distinct microhabitats generates correlations between morphological and ecological traits, and may increase both phenotypic and taxonomic diversity. However, some microhabitats exert unique selective pressures that act as a restraining force on macroevolutionary patterns of diversification. In this study, we use phylogenetic comparative methods to investigate the evolutionary outcomes of inhabiting the arboreal microhabitat in salamanders. We find that arboreality has independently evolved at least five times in Caudata and has arisen primarily from terrestrial ancestors. However, the rate of transition from arboreality back to terrestriality is 24 times higher than the converse. This suggests that macroevolutionary trends in microhabitat use tend toward terrestriality over arboreality, which influences the extent to which use of the arboreal microhabitat proliferates. Morphologically, we find no evidence for an arboreal phenotype in overall body proportions or in foot shape, as variation in both traits overlaps broadly with species that utilize different microhabitats. However, both body shape and foot shape display reduced rates of phenotypic evolution in arboreal taxa, and evidence of morphological convergence among arboreal lineages is observed. Taken together, these patterns suggest that arboreality has played a unique role in the evolution of this family, providing neither an evolutionary opportunity, nor an evolutionary dead end.

## 1 INTRODUCTION

Understanding how taxa respond evolutionarily to the microhabitats they utilize is of major interest in evolutionary biology. That differing microhabitats exert unique ecological selection pressures is axiomatic in biology, and species inhabiting distinct microhabitats often display phenotypic or functional differences resulting from adaptive diversification in those niches (Irschick, Meyers, Husak, & Galliard, 2008; Kaliontzopoulou, Carretero, & Adams, 2015; Marques & Nomura, 2015; Price, Friedman, & Wainwright, 2015). Indeed, the observed phenotypic differences among taxa utilizing distinct microhabitats provide support for the power of ecological selection and is often treated as *primae facae* evidence of the ecomorphological paradigm positing that morphological adaptation follows microhabitat‐specific performance (*sensu* Wainwright & Reilly, 1994; Winemiller, 1991). In vertebrates, well‐studied examples of ecomorphological trends include the phenotypic differences displayed by *Anolis* lizards occupying different vegetation strata (Losos, 1992; Losos, Jackman, Larson, de Queiroz, & Rodrígues‐Schettino, 1998; Mahler, Ingram, Revell, & Losos, 2013), the distinct body forms of temperate freshwater fishes utilizing benthic and limnetic habitats (Berner, Adams, Grandchamp, & Hendry, 2008; Jastrebski & Robinson, 2004; Robinson, Wilson, Margosian, & Lotito, 1993; Schluter & McPhail, 1992), and the recurring phenotypes of African lake cichlids in distinct ecological zones (Albertson & Kocher, 2001; Fryer & Iles, 1972; Rüber & Adams, 2001), among others. Finally, when viewed at broader evolutionary scales, differential microhabitat use is also expected to play a pervasive role in the ecology of adaptive radiations and the generation of biodiversity (Schluter, 2000; Mahler et al., 2013; also Wainwright, 2007).

Among vertebrates, amphibians are generally considered to be ecologically restricted due to their affinities to water, yet some lineages have diversified into a variety of microhabitats, including the arboreal niche (Duellman, 2005; Moen, Irschick, & Wiens, 2013; Wake, 1987). Arboreal habitats present particular challenges for their inhabitants, as they confer unique selective pressures on many aspects of their natural history, including foraging strategies, predator avoidance, locomotion, and osmoregulation (see Astley & Jayne, 2009; Cartmill, 1985; Hood & Tschinkel, 1990; Losos, 1990). As a consequence, it is often hypothesized that use of an arboreal habitat may restrain phenotypic or taxonomic diversification of the lineages that inhabit them (Alencar, Martins, Burin, & Quental, 2017). Despite this prediction, some arboreal anuran lineages display increased species diversification rates as compared to less arboreal groups (Moen & Wiens, 2017), suggesting that in some circumstances, the transition to an arboreal lifestyle can facilitate rather than restrict the generation of biodiversity.

In salamanders, species of the family Plethodontidae display an impressive diversity of microhabitat use, including species that are fully aquatic, terrestrial, fossorial, cave‐dwelling, saxicolous, and even arboreal (Blankers, Adams, & Wiens, 2012; McEntire, 2016; Petranka, 1998; Wake, 1987). McEntire (2016) recently revealed that nearly 100 species (over 40% of the family) utilize arboreal and vegetative habitats, over 60 of which use these habitats as their primary microhabitat type, emphasizing the potential ecological importance of this understudied microhabitat. These species were found in over 20 genera across the family, prompting McEntire (2016) to hypothesize that arboreality is either an ancestral trait in plethodontids or has evolved multiple times across the phylogeny. Further, several arboreal salamanders possess webbing on their hands and feet, or have prehensile tails, both of which are thought to be adaptations that facilitate climbing (see Alberch, 1981; Darda & Wake, 2015; Wake & Lynch, 1976). Blankers et al. (2012) investigated general body proportions including approximately 40% of plethodontid species and found little differentiation in mean body shape across microhabitats, but the full extent to which arboreality has influenced the evolution of body and foot shape of species across the entire family remains unknown.

**Figure 1:**
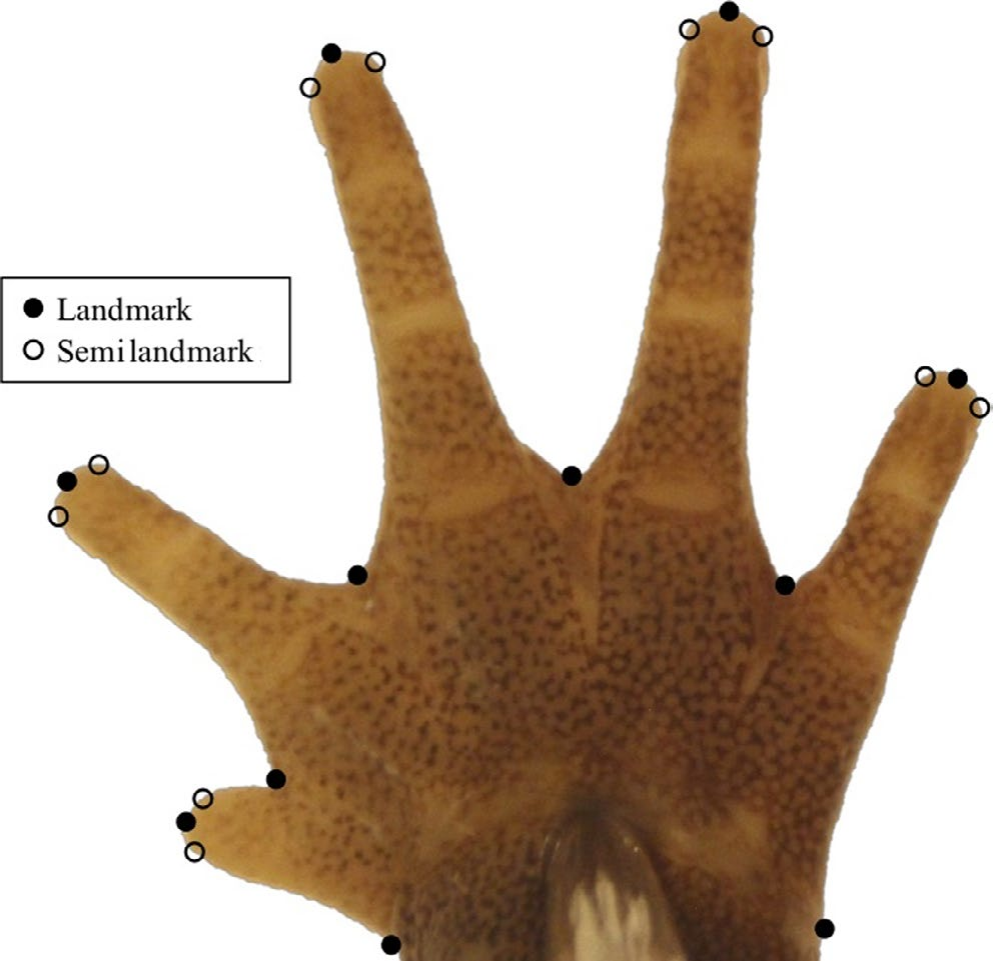
Landmark placement for foot shape analysis. Eleven landmarks and ten semilandmarks captured foot shape for each specimen. Missing landmarks were estimated using interpolation

Given the frequency with which plethodontids exploit arboreal microhabitats, the macroevolutionary consequences of arboreality have been surprisingly understudied in this group. To fill this void, we investigated the history of microhabitat use across salamanders in order to evaluate the extent to which use of the arboreal microhabitat influences patterns of morphological diversification. To accomplish this, we used a time‐calibrated molecular phylogeny for salamanders (Bonett & Blair, 2017) and estimated rates of microhabitat transitions across the evolutionary history of salamanders using data obtained from the literature. We then evaluated whether use of an arboreal microhabitat represents a single evolutionary event in plethodontids, or whether multiple transitions to this unique microhabitat have occurred. Additionally, we characterized body shape using linear measurements and foot shape using two‐dimensional geometric morphometrics (Figure 1) to test whether arboreal species display distinct patterns in their morphology, which would provide evidence of an arboreal phenotype. We also evaluated whether rates of phenotypic diversification were affected by microhabitat use and tested for the presence of phenotypic convergence across arboreal lineages. Finally, we estimated the ancestral microhabitat type at the root of Plethodontidae to shed light on the deeper evolutionary history of microhabitat use in the group. Historically, it has been assumed that plethodontids arose in an aquatic habitat (Beachy & Bruce, 1992; Dunn, 1926); however, recent life history data on salamander life cycles (Bonett & Blair, 2017) revealed that ancestral plethodontids displayed direct development. This observation leads to the intriguing hypothesis that the ancestral plethodontid may have been of terrestrial origin. We evaluate that possibility here.

## 2 MATERIALS AND METHODS

### 2.1 Phylogeny

**Figure 2:**
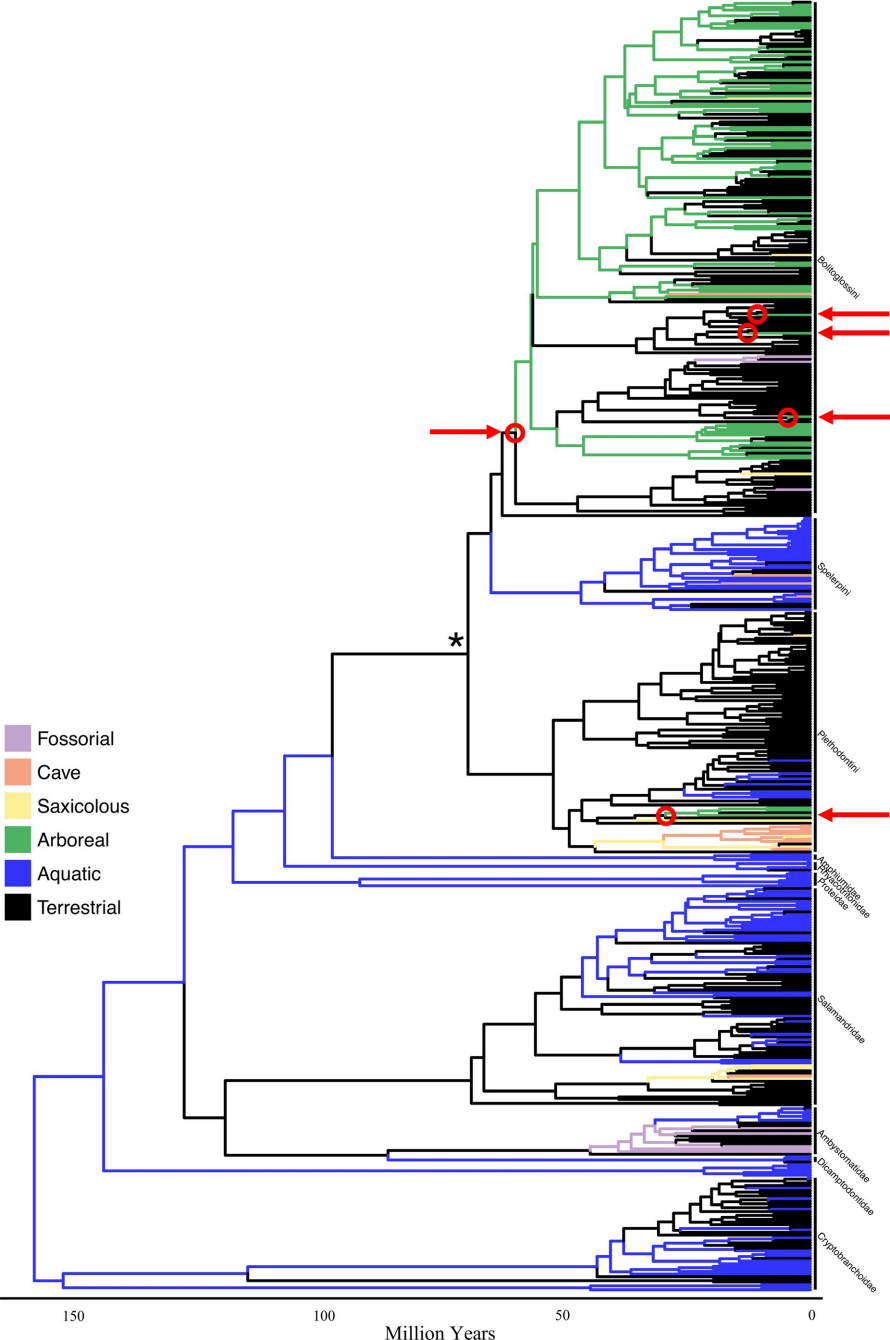
Time‐dated phylogeny for salamanders from Bonett and Blair (2017), pruned to the 495 species for which microhabitat data were available in this study. Tips are labeled using the 6‐M microhabitat classification scheme (see text), and branches are colored based on stochastic mapping node estimates. Red circles indicate likely transitions from terrestrial to arboreal microhabitats based on Bayesian stochastic mapping, and the root of Plethodontidae is marked with an asterisk. Red arrows assist in locating red circles

We used a multigene time‐calibrated phylogeny for Caudata from Bonett and Blair (2017) to evaluate our macroevolutionary hypotheses. This phylogeny included 516 taxa and was estimated from three mitochondrial and four nuclear genes using Bayesian approaches under a pure‐birth speciation prior on the tree topology and divergence times, an uncorrelated lognormal molecular clock, and 12 node calibrations based on Shen et al. (2016: for additional details see Bonett & Blair, 2017). Our primary analyses were based on the maximum clade credibility tree derived from a set of 1,000 posterior chronograms (Bonett & Blair, 2017). Specifically, we pruned the summary phylogeny of Bonett and Blair (2017) so that it contained only the species for which microhabitat data were available, resulting in a 495 species phylogeny (Figure 2). This included 327 species from the family Plethodontidae; approximately 71% of the recognized diversity for this clade. Likewise, we pruned each of the 1,000 dated trees in the posterior sample to match our species‐level data in order to perform sensitivity analyses with respect to phylogenetic uncertainty (see below).

### 2.2 Microhabitat classification

We characterized adult microhabitat use for 495 salamander species present on the phylogeny of Bonett and Blair (2017; Figure 2) using published literature and accounts from field observations. Most of the microhabitat data were obtained from AmphibiaWeb (2016), Blankers et al. (2012), IUCN (2010), McEntire (2016), Petranka (1998), Wake (1987), and Wake and Lynch (1976), and was corroborated by additional primary sources. For species not included in these broader surveys, microhabitat data were obtained from species accounts, species descriptions, and other natural history sources. Our classification procedures roughly followed that of previous authors (Blankers et al., 2012; McEntire, 2016; Wake & Lynch, 1976) and included the following microhabitat categories: arboreal (A), cave (C), fossorial (F), saxicolous (S), terrestrial (T), and aquatic (W). We included bromeliad dwellers in the arboreal category and moss mat dwellers in the terrestrial category, unless the moss mat was specified as arboreal. Those species commonly found on understory vegetation, such as ferns, were categorized as terrestrial.

Species that utilized more than one microhabitat during their adult lives (approximately 1/3 of the species in this study) were assigned both a primary and secondary microhabitat category. Microhabitat use for these species could theoretically be measured as a proportion of the species' life spent in each microhabitat, but these data were not available for this study for two reasons: there was considerable intraspecies variation in microhabitat use, and reliable proportion data of this sort were not available on a macroevolutionary scale. These considerations led us to employ six different microhabitat classification schemes (6‐M, 6‐L, 7‐M, 7‐L, 6‐McM, and 6‐McL) that were used to evaluate the robustness of our macroevolutionary inferences with respect to the limitations of this dataset. These six classification schemes represent two different approaches (majority‐rule and lenient; abbreviated as M and L, respectively) for each of three different biological considerations: our classification (6‐M and 6‐L), our classifications considering an independent semiaquatic microhabitat (7‐M and 7‐L), and McEntire's (2016) arboreal classifications (6‐McM and 6‐McL). All classification schemes are described in more detail below and are summarized in Table 1. Abbreviations represent the number of possible states for the scheme and the approach used.

**Table 1:** Microhabitats used in each classification scheme. Microhabitat abbreviations are arboreal (A), cave (C), fossorial (F), saxicolous (S), semiaquatic (SA), terrestrial (T), and aquatic (W). A brief description of each scheme is provided

Scheme	Microhabitats	Description
6‐M	T, W, A, C, F, S	Six microhabitats: majority‐rule designation
6‐L	T, W, A, C, F, S	Six microhabitats: lenient designation (sometime not T)
7‐M	T, W, A, C, F, S, SA	Seven microhabitats (semiaquatic added): majority‐rule
7‐L	T, W, A, C, F, S, SA	Seven microhabitats (semiaquatic added): lenient
6‐McM	T, W, A, C, F, S	Six microhabitats: majority‐rule, McEntire (2016) obligate arboreal
6‐McL	T, W, A, C, F, S	Six microhabitats: lenient, McEntire (2016) obligate + facultative arboreal

For our classification using a majority‐rule approach (6‐M), species were assigned to one of the six microhabitats (A, C, F, S, T, or W), based on the microhabitat in which they spend the majority of their adult life. The 6‐L scheme was more lenient in assigning species to nonterrestrial habitats (terrestrial being the dominant category), such that species occurring in both the terrestrial microhabitat and another microhabitat were assigned to the latter category (e.g., fossorial). Next, to account for the possibility that semiaquatic species might confer a unique ecological category distinct from both fully aquatic and fully nonaquatic species, we created a seventh category (semiaquatic: SA) and assigned species to microhabitats using both a seven category majority‐rule scheme (7‐M), and the more lenient scheme (7‐L), as in 6‐M and 6‐L, respectively. Finally, we evaluated variation in arboreal microhabitat designation by incorporating McEntire's (2016) arboreal classification scheme into the broader 6‐M and 6‐L classification schemes above. Specifically, we used McEntire's “obligate” arboreal classification to represent arboreal species in the 6‐M scheme above (6‐McM), and for a more lenient view of arboreality, we treated all of McEntire's arboreal designations (“obligate” and “facultative”) as arboreal taxa in the 6‐L classification scheme (6‐McL). In all cases, microhabitat was treated as an unordered, multistate character. Microhabitat classifications for all species across all classification schemes are available on Dryad (https://doi.org/10.5061/dryad.b554m44).

### 2.3 Morphology

To characterize morphology, we obtained seven linear measurements and images of the right hind foot using a digital camera with a macro lens from 3,169 adult specimens across 310 species of plethodontid salamanders included on the Bonett and Blair (2017) phylogeny. While prior body shape data were available, we chose not to use these in order to collect body shape and foot shape from the same specimens. We excluded several specimens from the foot shape dataset due to excessive foot damage, malformation, or lack of a fifth toe (i.e., all species in the genus *Batrachoseps*), leaving 2,810 usable specimens across 288 species. Sample sizes varied between 1 and 33 specimens per species (mean = 10.22) for body shape and between 1 and 32 specimens per species (mean = 9.76) for foot shape, which was determined by the availability of specimens in museum collections. Generally, within‐species sexual size dimorphism in plethodontids is small as compared to size differences between species (Petranka, 1998). Therefore, we did not perform separate analyses on each sex, but rather combined all specimens for our analyses, as in previous macroevolutionary studies (Blankers et al., 2012).

To quantify body shape for each specimen, we measured snout–vent length (SVL), tail length (TL), head length (HL), body width (BW), snout‐eye distance (SE), forelimb length (FLL), and hind limb length (HLL) as these measures are considered to capture the major variation in general body shape (Adams, Berns, Kozak, & Wiens, 2009; Bonett & Blair, 2017, see Blankers et al., 2012 for measurement details). For the specimens with damaged anatomical components, measurements were only taken from the regions that were intact. In such cases, statistical imputation via multivariate multiple regression was used to estimate the missing values. Of the 21,812 total measurements, only 1.97% (430) required imputation. For each species, mean values for all linear measurements were obtained, and body proportions were calculated by dividing all variables by body size (SVL). This resulted in a set of shape ratios (*sensu* Mosimann, 1970) which were then log‐transformed and matched to the phylogeny of Bonett and Blair (2017) for subsequent phylogenetic comparative analyses.

Foot shape was characterized using two‐dimensional landmark‐based geometric morphometrics (Adams, Rohlf, & Slice, 2013; Bookstein, 1991), digitizing 11 landmarks and 10 semilandmarks on each foot photograph. Landmarks correspond to the tips of the toes and the minimal extent of webbing between the toes, and semilandmarks were placed along the edge of the toe tip to capture toe width (Figure 1). Missing landmarks and semilandmarks were estimated using thin‐plate spline interpolation based on conspecifics. For specimens without complete conspecific specimens from which to estimate the missing landmarks, we used sister species specimens based on the Bonett and Blair maximum clade credibility tree (2017). Of 59,010 total landmarks across 2,810 specimens, only 1.63% (959) required interpolation. We obtained species means by aligning specimens within each species using a generalized Procrustes analysis to remove nonshape variation of position, rotation, and scale, allowing semilandmarks to slide between the bracketing landmarks by minimizing bending energy. We then aligned these means with the same procedure to use in all subsequent analyses. All morphological data, including a list of species and specimens used in this study, are available in Dryad (https://doi.org/10.5061/dryad.b554m44).

### 2.4 Phylogenetic comparative analyses

We used both maximum likelihood (ML) methods and Bayesian stochastic mapping to characterize the evolutionary history of microhabitat use across the phylogeny. First, we compared the empirical fit of the microhabitat data to the phylogeny under three evolutionary models (equal rates: ER, symmetric: Sym, and all rates different: ARD) and identified the optimal model of discrete character evolution using AIC. We then used the matrix of transition rates (Q‐matrix: *sensu* Pagel, 1999) obtained under the optimal model (ARD, see Results) to estimate ancestral microhabitat use under a ML framework. Additionally, we conducted Bayesian stochastic character mapping (Bollback, 2006; Huelsenbeck, Nielsen, & Bollback, 2003) to estimate shifts in microhabitat use across the phylogeny and to evaluate transition rates among microhabitat categories. Here we generated 1,000 stochastic maps across the maximum clade credibility tree, using the Q‐matrix calculated using maximum likelihood under the optimal ARD model (see Results). These stochastic maps were then summarized to obtain estimates of microhabitat use at each node of the phylogeny, including at the root of Plethodontidae, and to provide estimates of the number of evolutionary transitions between microhabitat categories.

To evaluate morphological trends in body shape and foot shape, we employed a variety of comparative methods to examine shape mean, rate of evolution, and convergence. First, we performed a multivariate phylogenetic analysis of variance (phylogenetic ANOVA: *sensu* Adams, 2014a; Adams & Collyer, 2018; Garland, Dickerman, Janis, & Jones, 1993) to determine whether species utilizing distinct microhabitat types differ in mean shape. Residual randomization permutation procedures (Collyer, Sekora, & Adams, 2015) were used to evaluate model significance. Pairwise comparisons were then performed using Euclidean distances between phenotypic means for each microhabitat, which were statistically evaluated using the same permutation procedure. Next, patterns of phenotypic variation were visualized in a phylomorphospace (*sensu* Rohlf, 2002; Sidlauskas, 2008), where the phenotypic data were rotated via a principal components analysis (PCA) and the phylogeny was superimposed. To evaluate the extent to which arboreal species have converged morphologically, we quantified two convergence measures recently proposed by Stayton (2015). The first (*C*_1_) characterizes the magnitude of morphological convergence in focal extant taxa relative to the maximal divergence in their ancestral values, with larger values representing a greater degree of phenotypic convergence. The second (*C*_5_) measures the frequency of convergence to a particular region of morphospace, estimated by the number of focal lineages whose evolution transects the boundary region defined by the focal taxa. Both measures were evaluated via phylogenetic simulation under Brownian motion (see Stayton, 2015). We also quantified the rate of multivariate phenotypic evolution across species within each microhabitat category (*sensu* Adams, 2014b) and compared these evolutionary rates statistically using Brownian motion simulations. Because cave species display evolutionary ontogenetic convergence of foot shape across species related to climbing (Adams & Nistri, 2010), we also tested whether arboreal species' foot shapes have allometrically converged with cave‐dwelling species' foot shape. We tested this using a permutation procedure derived from Adams and Nistri (2010).

To account for uncertainty in microhabitat designations, each analysis was repeated using the different microhabitat classification schemes described above (Table 1). Likewise, to account for phylogenetic uncertainty, all analyses were repeated on the set of chronograms from the posterior distribution of Bonett and Blair (2017) to estimate 95% confidence intervals on values obtained from the maximum clade credibility tree. All analyses were performed in R 3.5.0 (R Core Team, 2018), using the packages corHMM (Beaulieu, Oliver, & O'Meara, 2017), phytools (Revell, 2018), convevol (Stayton, 2017), and geomorph (Adams, Collyer, & Kaliontzopoulou, 2018; Adams & Otarola‐Castillo, 2013).

## 3 RESULTS

**Figure 3:**
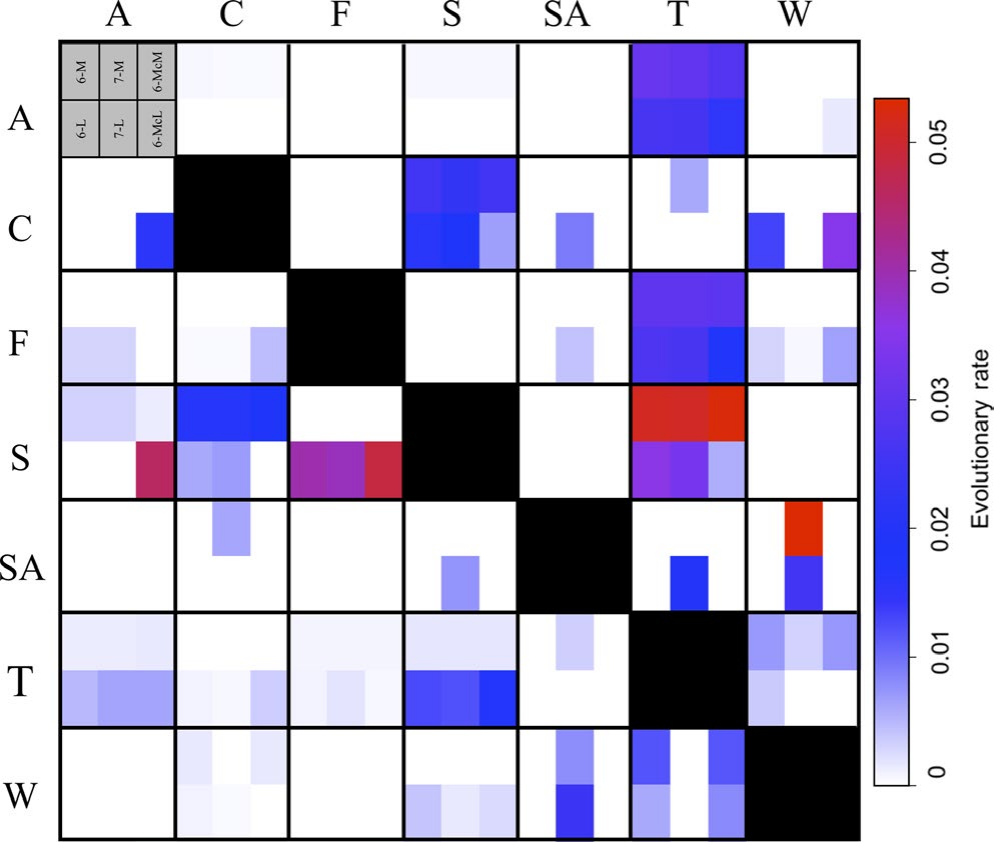
Heat map of Q‐matrix representing transition rates between microhabitat categories for Bayesian stochastic mapping. As is convention for Q‐matrices, rows represent the microhabitat type of origin, while columns represent the ending microhabitat type for each pairwise transition rate. Diagonals have been omitted. Each cell in the six‐by‐six Q‐matrix is divided into six subcells representing the six different classification schemes. The top three subcells represent the majority‐rule classification schemes (6‐M, 7‐M, and 6‐McM, left to right) and the bottom three cells represent the lenient classification schemes (6‐L, 7‐L, and 6‐McL, left to right). Microhabitat abbreviations are arboreal (A), cave (C), fossorial (F), saxicolous (S), semiaquatic (SA), terrestrial (T), and aquatic (W)

**Figure 4:**
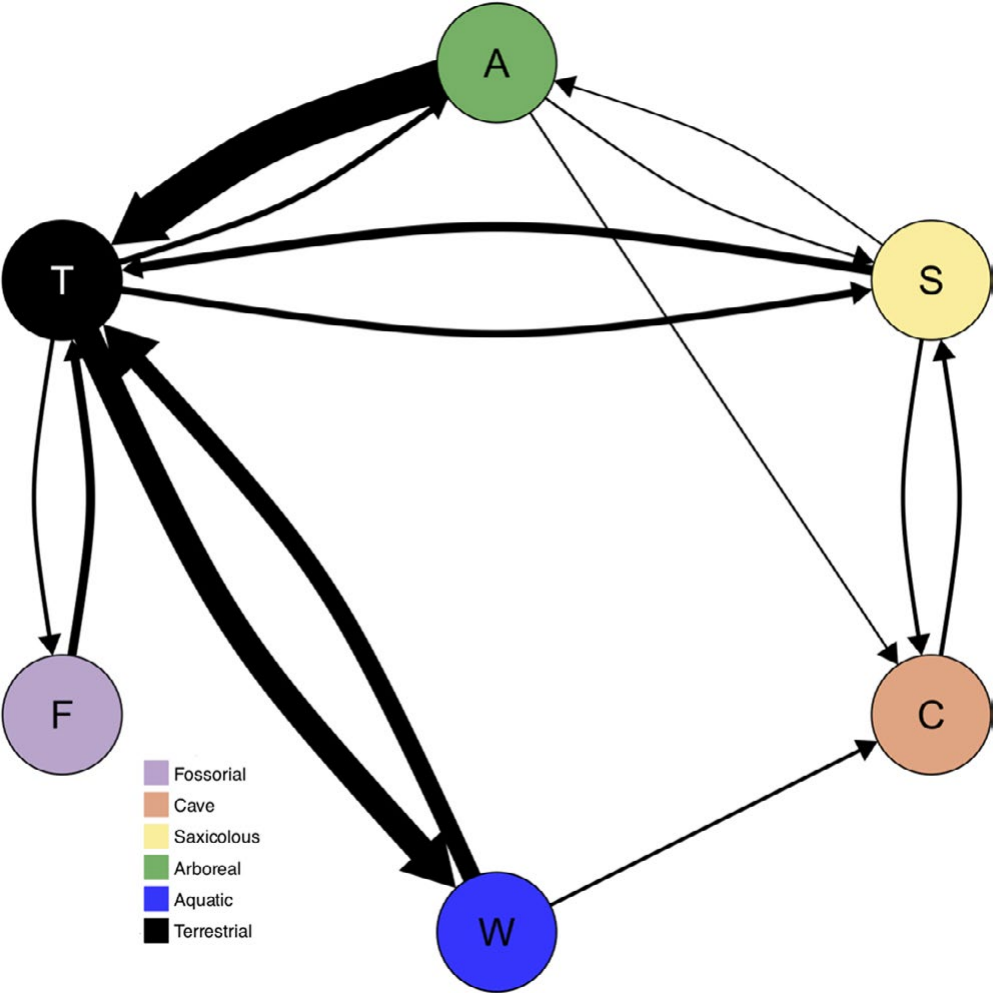
Number of transitions between microhabitat categories using the 6‐M microhabitat classification and Bayesian stochastic character mapping. Thickness of arrows is proportional to the number of transitions with a high of 63.2 (A to T) and a low of 0.763 (S to A). For exact numbers of transitions under all classification schemes, see Appendix S2

The all rates differ (ARD) evolutionary model provided the best fit of the microhabitat data to the phylogeny (∆AIC > 27.45; Appendix S1). Examination of the resulting Q‐matrix (Figure 3) revealed that transition rates to and from arboreality differed. Specifically, transition rates from terrestriality to arboreality were nearly 24 times lower than the converse (*q*_t→a_ = 0.0013; *q*_a→t_ = 0.0306: Figure 3), and transition rates to and from arboreality with other microhabitat categories (e.g., cave, fossorial, and aquatic) were zero (Figure 3). Results from stochastic mapping were consistent with these observations and provided support for multiple origins of arboreality in plethodontids (Figure 2). Further, we observed that the evolution of arboreality occurred primarily from a terrestrial ancestor (Figure 4). Though estimates varied slightly depending on which microhabitat classification scheme was evaluated, at least five independent transitions to arboreality were inferred by the data, thereby rejecting the hypothesis that arboreality evolved only once in the group (Figure 2; Appendix S2). We also estimated over 60 transitions away from arboreality (Figure 4) primarily to the terrestrial microhabitat, which is consistent with the observed high transition rate from arboreal to terrestrial microhabitats. Notably, one transition to arboreality was deeply nested within the plethodontid phylogeny near the root of neotropical Bolitoglossini salamanders (Figure 2). This was not surprising, as most arboreal species are members of this lineage. Additionally, most transitions from arboreality to terrestriality were observed within the neotropical Bolitoglossini (Figure 2). The remaining transitions to arboreality were more recent and were scattered throughout the plethodontid phylogeny. Finally, both maximum likelihood and Bayesian ancestral state estimation supported a nonaquatic ancestor for the root of Plethodontidae and did so with high support (6‐M ML: 97.5%; 6‐M Bayesian: 97%; Figure 2; Appendix S3). All results were consistent when evaluated across the microhabitat classification schemes and across the set of 1,000 posterior chronograms, indicating that these macroevolutionary inferences were robust to variation in microhabitat designation as well as to phylogenetic uncertainty (Appendix S3).

**Figure 5:**
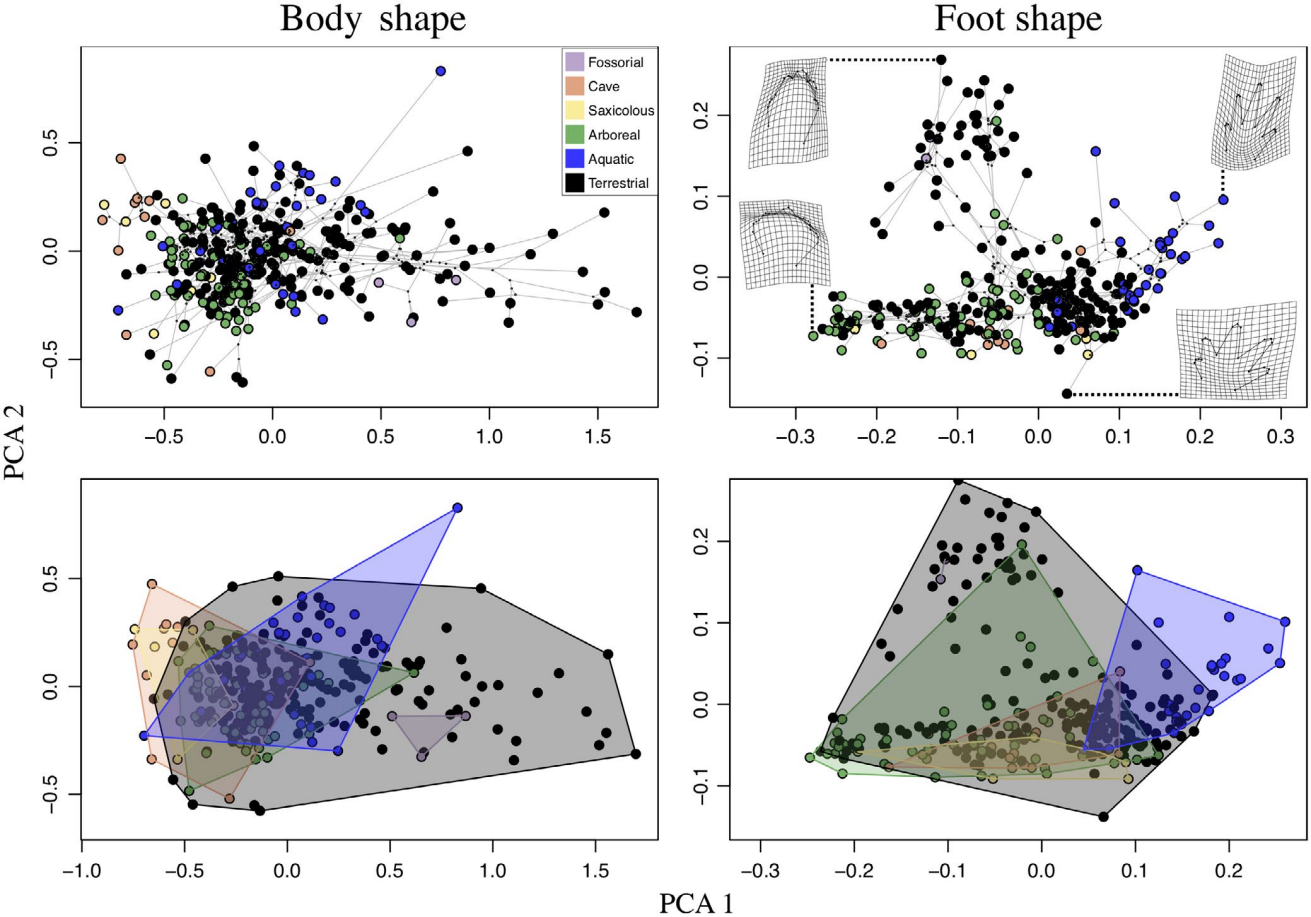
Phylomorphospace representing dispersion among species in their general body proportions and foot shape. Top panels show all species means colored by microhabitat classification (6‐M) for body shape and foot shape. The bottom panels represent the convex hulls defined by all species in a microhabitat type using 6‐M with notable overlap between the arboreal and other microhabitat type convex hulls. The first two axes of phylomorphospace describe 89.19% and 79.27% of the total variation for body and foot shape, respectively (PCA1_Body_ = 73.86%; PCA2_Body_ = 15.33%; PCA1_Foot_ = 52.23%; PCA2_Foot_ = 27.04%)

When evaluating morphological trends, most results were consistent across body and foot shape and are thus presented together. First, the phylogenetic ANOVAs revealed no differences in either general body proportions or foot shape among microhabitat groups (body shape: *R* ^2^ = 0.0237, *F* = 1.475, *Z* = 1.074, *p* = 0.143 NS; foot shape: *R* ^2^ = 0.0229, *F* = 1.322, *Z* = 0.9263, *p* = 0.178 NS), and pairwise comparisons similarly revealed that arboreal species did not differ phenotypically from species utilizing other microhabitat types (results not shown). Our body shape results were consistent with previous findings based on a reduced set of taxa (Blankers et al., 2012) and suggest that arboreal salamanders do not exhibit a unique arboreal phenotype in terms of their general body proportions. Patterns in phylomorphospace reaffirmed these statistical findings for both traits, as a broad overlap among microhabitat groups was detected, thus revealing that arboreal taxa do not occupy a distinct region of morphospace for either body shape or foot shape (Figure 5). This finding was consistent across all microhabitat classification schemes used in this study.

However, when levels of morphological convergence were evaluated, arboreal species did display some degree of phenotypic consistency (body shape: *C*_1_ = 0.208; *p* = 0.03; *C*_5_ = 11; *p* = 0.01; foot shape: *C*_1_ = 0.095; *p* = 0.87; *C*_5_ = 10; *p* = 0.01). In particular, *C*_5_ showed that these species occupy the same general region of morphospace more often than is expected by chance under Brownian motion for both traits, though this region was not uniquely occupied by arboreal taxa. Similarly, *C*_1_ revealed that, on average, arboreal species displayed a 21% reduction in their body shape differences as compared to the maximal spread of their ancestors. Therefore, while the typical body form and foot shape of arboreal species was not distinct from that of species utilizing other microhabitat types, arboreal species exhibited greater morphological constancy than expected. Finally, rates of phenotypic evolution differed significantly among microhabitat groups. For body shape, arboreal species displayed a rate of phenotypic evolution that was 2.21 times slower than that observed among terrestrial species ($\sigma_{A}^{2}=4.72\times 10^{-4}$ vs. $\sigma_{T}^{2}=1.04\times 10^{-3}$; *p* = 0.001; Appendix S4), and 4.74 times slower than that found among aquatic species ($\sigma_{A}^{2}=4.72\times 10^{-4}$ vs. $\sigma_{W}^{2}=2.24\times 10^{-3}$; *p* = 0.001; Appendix S4). Likewise, foot shape in arboreal species displayed a rate of phenotypic evolution that was 1.82 times slower than that observed among terrestrial species ($\sigma_{A}^{2}=5.39\times 10^{-6}$ vs. $\sigma_{T}^{2}=9.81\times 10^{-6}$; *p* = 0.001; Appendix S4) and 5.28 times slower than that found among aquatic species ($\sigma_{A}^{2}=5.39\times 10^{-6}$ vs. $\sigma_{W}^{2}=2.85\times 10^{-5}$; *p* = 0.001; Appendix S4). *C*_1_ and *C*_5_ analyses were too computationally intensive to repeat over the 1,000 posterior chronograms and all classification schemes but were evaluated across classification schemes 6‐M and 6‐L. While *C*_5_ for body shape was robust to microhabitat classification, *C*_5_ for foot shape and C_1_ for both morphological traits were not (*C*_5_: *p*_Foot6‐L_ = 0.51; *C*_1_: *p*_Body6‐L_ = 0.34; *p*_Foot6‐L_ = 0.91). Although some measures of convergence were not robust to microhabitat designation, all other results indicate that our macroevolutionary inferences are robust to variation in microhabitat designation as well as to phylogenetic uncertainty.

Our final analysis of foot morphology tested whether the foot shapes of arboreal species allometrically converged with those of cave‐dwelling species. Using a permutation‐based procedure adopted from Adams and Nistri (2010), we found significant allometric convergence in foot shape (*D*_small_ − *D*_large_ = 0.130; *p* = 0.01). Specifically, as arboreal and cave species foot sizes increase, they converge on the same shape more so than is expected by chance. This pattern was largely robust to classification scheme (see Appendix S5).

## 4 DISCUSSION

How organisms respond to selection pressures across differing microhabitats is a major topic in evolutionary biology. In this study, we characterized the evolutionary history of microhabitat use among salamanders to elucidate how exploiting the arboreal niche has influenced the diversification of this group. Using both maximum likelihood and Bayesian ancestral state estimation methods, we inferred that arboreality has evolved independently at least five times in plethodontid salamanders. Our data strongly indicate that across these transitions, arboreality evolved primarily from terrestrial ancestors, and transition rates from arboreality to terrestriality were considerably higher than the converse. Our analyses also strongly supported a terrestrial ancestor at the root of Plethodontidae. Morphologically, arboreal salamanders’ body shapes and foot shapes were not different from their terrestrial counterparts, providing little evidence for the existence of a distinct arboreal phenotype. However, there was evidence of body and foot shape convergence among arboreal salamanders, and arboreal taxa displayed reduced rates of phenotypic evolution as compared to both terrestrial and aquatic salamander species. Finally, we found significant allometric convergence of arboreal and cave‐dwelling foot shape as foot size (centroid size) increases. Overall, our findings provide several key insights into the evolution of arboreality in salamanders regarding the unique evolutionary role arboreality plays in this group as compared to nonarboreal taxa.

Previous investigations of arboreality in other clades have tended to conform to one of two macroevolutionary interpretations. First, arboreality appears to provide an evolutionary opportunity for some lineages (i.e., anurans, Moen et al., 2013, *Anolis* lizards, Losos, 2009; ground beetles, Ober, 2003). These patterns are evidenced by the presence of many extant arboreal species, several independent transitions toward arboreality, few transitions away from arboreality, and increased rates of morphological evolution converging on a distinct arboreal phenotype. Alternatively, in some lineages, arboreality can act as a restraining force on subsequent diversification and is thus seen as an evolutionary constraint (i.e., vipers, Alencar et al., 2017). Such patterns are observed when clades display few extant arboreal species, few transitions to arboreality, few transitions away from arboreality, no distinct arboreal phenotype, and reduced rates of phenotypic evolution. Additionally, these two scenarios are often accompanied by patterns of increased or decreased species diversification rates, respectively, although such macroevolutionary patterns were not identified in this study.

In contrast to previous studies, our results suggest that the arboreal microhabitat plays a different role in the evolution of Plethodontidae that does not coincide perfectly with either of these perspectives, suggesting that our findings require a more nuanced interpretation. In particular, our results provide mixed support for both scenarios. For instance, our results confirmed McEntire's (2016) observation that a substantial proportion of extant plethodontid species are arboreal, and we found several independent transitions to arboreality. These results align with the first scenario describing arboreality as an evolutionary opportunity. However, we also observed many more reversals to terrestriality than successful colonizations of the arboreal niche from a terrestrial ancestor (Figures 2 and 4), mirrored by the substantially higher rate of transitions out of arboreality than toward arboreality. This latter pattern suggests that macroevolutionary trends in plethodontid microhabitat use favor terrestriality over arboreality, which does not suggest that arboreality is an evolutionary opportunity for plethodontids. Instead, these results indicate that arboreality may act as an evolutionarily transitory state in the family Plethodontidae, such that arboreality has evolved several times and has persisted in some lineages, but most lineages readily revert to terrestrial life.

The results from our morphological analyses also show mixed support for the evolutionary scenarios described in other taxa. Specifically, we observed that arboreal species are not phenotypically distinct from their terrestrial counterparts, yet we identified evidence of phenotypic convergence and reduced rates of phenotypic evolution in both body shape and foot shape in arboreal salamanders. These patterns suggest that, while there is not a unique arboreal phenotype, use of the arboreal microhabitat does impose some selective forces for a common phenotype. Further, just as cave‐dwelling species experience ontogenetic convergence toward a climbing foot shape (Adams & Nistri, 2010), the allometric convergence of foot shape between cave and arboreal species observed in this study implicate selective forces related to climbing that may be common to utilizing both of these microhabitats. Thus, our observations do not align precisely with either of the two evolutionary scenarios described in other taxa. Additionally, if arboreality acts as an evolutionary transitory state, as we have posited, our results show that transitions between terrestrial and arboreal life are not limited by morphology. Rather, some other mechanism is hypothesized to drive the high rate and frequency of transitions away from arboreality. We were unable to define this precise mechanism in the present study, although investigations into intraspecific competition or abiotic conditions could provide further insight in this regard.

Perhaps, the most striking result from this study is the lack of a distinct foot shape for arboreal species. Previous research has demonstrated that some arboreal species have anatomical features associated with climbing, such as webbing on their hands and feet (Alberch, 1981; Wake, 1987; Wake & Lynch, 1976) and tarsal rearrangements that increase the surface area of the foot (Wake, 1991). Further, cave‐dwelling species that climb extensively display unique patterns of foot morphology (Adams, Korneisel, Young, & Nistri, 2017; Adams & Nistri, 2010). However, our results demonstrate that the broad, webbed foot shape often considered to be an arboreal specialization is not unique to, nor necessary for, arboreal species. Indeed, prior developmental work has shown a common underlying mechanism for the evolution of foot webbing in both arboreal and nonarboreal tropical taxa (Jaekel & Wake, 2007), which may explain some of the patterns we observed. Further, our results align with a recent study demonstrating that clinging ability is not unique to arboreal species (M. K. O'Donnell, personal communications). These results suggest that many terrestrial species are morphologically capable of occupying the arboreal microhabitat, and transitions away from arboreality are not driven by biomechanical limitations.

One possible limitation of our study is that there may be other traits besides foot shape and general body proportions that convey a selective advantage in arboreal taxa. For example, prehensile tails are used to aid in arboreal locomotion in several species (Darda & Wake, 2015). However, as prehensile tails have been observed in several nonarboreal species as well (i.e., cave‐dwelling *Eurycea lucifuga*, Petranka, 1998; terrestrial *Phaeognathus hubrichti*, Blair, 1967), we think it unlikely that this trait would show markedly different patterns than those presented in this paper. Further, because the degree of tail prehensility is not characterized for many taxa, the effects of this trait on macroevolutionary patterns of diversification remain unknown.

Our study also confirmed that primary use of the arboreal microhabitat in species outside of the neotropical Bolitoglossini group is relatively rare. Arboreality is observed in several species of the temperate genus *Aneides* (McEntire, 2016; Petranka, 1998), with one species, *A. vagrans* occasionally found in the canopy over 70 meters above the forest floor (Spickler, Sillett, Marks, & Welsh, 2006). *Aneides* is the only temperate lineage with species that obligately occupy arboreal habitats. On the other hand, arboreal species are found in at least 19 genera of neotropical salamanders, many of whom utilize specific components of the arboreal microhabitat (e.g., bromeliads; see Wake, 1987; Wake & Lynch, 1976). All classification schemes investigated in this study found a transition to arboreality at the root of this neotropical clade (Figure 2), and this early tropical transition toward arboreality may have been followed by subdivision of the arboreal microhabitat into smaller ecological niches (e.g., bromeliad, under bark, canopy). While many neotropical species have since reacquired use of terrestrial microhabitats, arboreality remains pervasive within the group, suggesting that arboreality and subdivisions of the arboreal microhabitat may have played an important role in the diversification of plethodontids in the neotropics (see also Wake, 1987).

Although not the focus of this paper, the analyses quantifying evolutionary rate revealed substantially higher rates of phenotypic evolution in aquatic species for both body shape and foot shape. This pattern was first described by Bonett and Blair (2017) across the entire order Caudata, where fully aquatic species displayed an increased rate of body shape and vertebral column diversification compared to fully terrestrial or semiaquatic species. Our results are therefore consistent with these observations and show that this interesting pattern holds when looking within a large clade with relatively few aquatic species.

Finally, our analyses reveal strong support for a terrestrial ancestor at the root of plethodontid salamanders (Figure 2) regardless of microhabitat classification scheme. To our knowledge, our study is the first to utilize a phylogenetic framework to thoroughly elucidate evolutionary trends of microhabitat use in plethodontids and suggests that the transition from an aquatic to a terrestrial lifestyle occurred at the earliest stages of this incipient salamander radiation. However, the observation that early plethodontids were likely terrestrial stands in direct contrast with the dominant, and long‐standing, hypothesis that plethodontids originated from an aquatic (and biphasic) ancestor that inhabited fast‐flowing mountain streams in southeastern North America (Beachy & Bruce, 1992; Wilder & Dunn, 1920). In addition, this Wilder–Dunn (1920) hypothesis also posited that the evolution of lunglessness, a trait shared by all plethodontid species, but which is otherwise exceedingly rare in vertebrates, occurred as a rheotropic adaptation to larval life (Beachy & Bruce, 1992). While the evolution of lunglessness remains a major macroevolutionary paradox, several lines of evidence render the Wilder–Dunn (1920) hypothesis incompatible with current observations. For instance, recent phylogenetic analyses of life cycle evolution have convincingly demonstrated that the ancestor of Plethodontidae exhibited direct development, rather than a biphasic lifestyle that included an aquatic larval stage (Bonett & Blair, 2017; also Chippendale, Bonett, Baldwin, & Wiens, 2004). Further, because all extant direct developing plethodontids utilize terrestrial microhabitats, having a direct developing ancestor implies that terrestriality was basal for the clade as well. Our macroevolutionary analyses confirm this prediction and provide strong support for a terrestrial origin of plethodontid salamanders (Figure 2). Thus, our results, in combination with those of Bonett and Blair (2017), provide convincing evidence rejecting the two primary assumptions of the Wilder–Dunn hypothesis. While other hypotheses for the evolution of lunglessness have been proposed (Reagan & Verrell, 1991), none have been tested empirically. Therefore, at present, phylogenetic patterns in both life history and microhabitat evolution suggest that the evolution of lunglessness in plethodontid salamanders is likely not a rheotropic adaptation to an aquatic lifestyle, but instead requires an alternative explanation that, to date, has not been fully examined empirically.

## CONFLICT OF INTEREST

None declared.

## AUTHOR CONTRIBUTIONS

EKB and DCA conceived of the study, EKB collected the data and conducted all analyses, EKB and DCA contributed equally to the writing of the manuscript.

## Data Availability

All data in this manuscript are available on DRYAD (https://doi.org/10.5061/dryad.b554m44).
