# Supplementary material for: Macroevolution of arboreality in salamanders
Source: Ecol Evol. 2019 May 26;9(12):7005–16. doi: 10.1002/ece3.5267 (PMC6662381; doi:10.1002/ece3.5267)
Supplement: Supplementary file 1 [file ECE3-9-7005-s001.docx]

Appendix S1: ML model comparison results for all classification schemes.

Table S1. Models include Equal Rates (ER), Symmetrical (Sym), and All Rates Differ (ARD). 95% confidence intervals were calculated by running all analyses across the posterior chronograms. In all cases, ARD was the best fit model.

| **Classification** | **Model** | **AIC** | **∆AIC** | **CI** |
| --- | --- | --- | --- | --- |
| 6-M | ER | 1011.77 | 172.54 | (1011.184, 1012.365) |
|  | Sym | 913.44 | 74.21 | (912.969, 913.917) |
|  | ARD | 839.23 | 0 | (838.863, 839.589) |
| 6-L | ER | 1243.00 | 133.05 | (1242.513, 1243.485) |
|  | Sym | 1153.34 | 43.39 | (1152.971, 1153.704) |
|  | ARD | 1109.95 | 0 | (1109.584, 1110.314) |
| 7-M | ER | 1141.72 | 174.53 | (1141.059, 1142.374) |
|  | Sym | 1052.48 | 85.29 | (1051.954, 1053.002) |
|  | ARD | 967.19 | 0 | (966.789, 967.602) |
| 7-L | ER | 1489.07 | 195.05 | (1488.541, 1489.604) |
|  | Sym | 1341.69 | 47.67 | (1341.286, 1342.086) |
|  | ARD | 1294.02 | 0 | (1293.634, 1294.414) |
| 6-McM | ER | 1034.61 | 182.50 | (1034.216, 1035.005) |
|  | Sym | 925.93 | 73.82 | (925.569, 926.292) |
|  | ARD | 852.11 | 0 | (851.690, 852.522) |
| 6-McL | ER | 1250.50 | 106.78 | (1249.976, 1251.026) |
|  | Sym | 1171.17 | 27.45 | (1170.786, 1171.551) |
|  | ARD | 1143.72 | 0 | (1143.336, 1144.113) |

Appendix S2: Stochastic mapping results estimating number of transitions towards arboreality across all classification schemes.

Table S1. Microhabitat classifications include cave (C), fossorial (F), saxicolous (S), semi-aquatic (SA), terrestrial (T), and aquatic (W). Ranges were generated from stochastic mapping analyses using the consensus tree and the 1000 posterior chronograms. All estimates averaging at least 1 transition are bolded. The set of stochastic maps with the fewest number of transitions towards arboreality was calculated using the 6-M and 7-M classification schemes. In both cases, at least 95% of the stochastic maps estimated 5 or more transitions. Notably, classification schemes 6-L, 7-L, and 6-McL have much higher estimates for the number of transitions towards arboreality. This is due to the more lenient rules used for classifying species as arboreal resulting in far more species with that microhabitat designation. The 6-L, 7-L, and 6-McL classifications include species in the arboreal category that are only partially arboreal. Further, the 6-McL classification includes many species as arboreal that occasionally climb on vegetation, rather than regularly inhabiting trees (see McEntire 2016).

|  | 6-M | 6-L | 7-M | 7-L | 6-McM | 6-McL |
| --- | --- | --- | --- | --- | --- | --- |
| C | 0 | 0 | -0.019-0.019 | 0 | -0.108-0.108 | **17.662-17.662** |
| F | 0 | **2.180-2.280** | -0.123-0.123 | **2.656-2.684** | 0 | **21.138-21.166** |
| S | 0.517-0.597 | 0 | 0.127-0.299 | 0 | -0.113-0.121 | 0 |
| SA | - | - | 0 | 0 | - | - |
| T | **7.6658-7.813** | **15.333-15.611** | **8.101-8.311** | **17.421-17.581** | **8.859-8.979** | **15.966-16.104** |
| W | 0 | 0 | 0 | 0 | -0.076-0.076 | 0 |

Appendix S3: Ancestral state reconstruction of microhabitat type at the root of Plethodontidae using maximum likelihood (ML) and Bayesian analyses (stochastic mapping; SM).

Table S1. Microhabitat classification types include arboreal (A), cave (C), fossorial (F), saxicolous (S), semi-aquatic (SA), terrestrial (T), and aquatic (W). Confidence intervals were generated by running the analyses across the 1000 posterior chronograms. The microhabitat type with the highest support for each analysis is bolded. All analyses support a non-aquatic ancestor with the exception of the 7-L classification scheme, which instead supports a semi-aquatic ancestor for Plethodontidae. Since the 7-M classification scheme provides almost no support for a semi-aquatic ancestor, the difference in results between the 7-M and 7-L analyses is most likely explained by the species that switch from T to SA between the two classification schemes. These species are those that spend most of their time in terrestrial microhabitats but occasionally occupy aquatic microhabitats. Thus, this result suggests that the ancestor for this family was primarily non-aquatic, but may have occasionally used aquatic habitats, which is in agreement with results from all other classification schemes.

| **Method** | **Scheme** | **A** | **C** | **F** | **S** | **SA** | **T** | **W** |
| --- | --- | --- | --- | --- | --- | --- | --- | --- |
| ML | **6-M** | 0.399±0.031 | 0.006±0.009 | 0.001±0.002 | 0.053±0.015 |  | **0.515±0.031** | 0.025±0.010 |
| SM |  | 0.415±0.031 | 0.005±0.005 | 0.000±0.002 | 0.048±0.018 |  | **0.498±0.031** | 0.030±0.009 |
| ML | **6-L** | 0.046±0.014 | 0.011±0.009 | 0.153±0.018 | 0.112±0.019 |  | **0.594±0.031** | 0.084±0.022 |
| SM |  | 0.033±0.016 | 0.008±0.005 | 0.153±0.015 | 0.096±0.020 |  | **0.604±0.030** | 0.106±0.018 |
| ML | **7-M** | 0.364±0.030 | 0.003±0.006 | 0.001±0.002 | 0.022±0.014 | 0.001±0.003 | **0.584±0.031** | 0.024±0.009 |
| SM |  | 0.364±0.029 | 0.007±0.008 | 0.002±0.004 | 0.032±0.020 | 0.002±0.004 | **0.565±0.031** | 0.028±0.009 |
| ML | **7-L** | 0.001±0.005 | 0.018±0.007 | 0.042±0.014 | 0.079±0.017 | **0.662±0.031** | 0.048±0.022 | 0.149±0.021 |
| SM |  | 0.000±0.025 | 0.019±0.007 | 0.040±0.009 | 0.068±0.019 | **0.675±0.021** | 0.048±0.031 | 0.150±0.012 |
| ML | **6-McM** | 0.342±0.021 | 0.002±0.013 | 0.002±0.002 | 0.020±0.005 |  | **0.606±0.015** | 0.028±0.009 |
| SM |  | 0.343±0.022 | 0.001±0.018 | 0.003±0.001 | 0.021±0.005 |  | **0.605±0.012** | 0.027±0.005 |
| ML | **6-McL** | 0.038±0.020 | 0.008±0.017 | 0.137±0.024 | 0.154±0.026 |  | **0.631±0.043** | 0.032±0.029 |
| SM |  | 0.032±0.015 | 0.007±0.005 | 0.141±0.016 | 0.149±0.019 |  | **0.638±0.030** | 0.033±0.018 |

Appendix S4: Robustness analyses of evolutionary rate comparisons across classification schemes.

Table S1. Pairwise comparisons of body shape evolutionary rates across different microhabitat types for 6-M classification scheme on the consensus phylogeny. Microhabitat classifications include arboreal (A), cave (C), fossorial (F), saxicolous (S), terrestrial (T), and aquatic (W). Pairwise rate ratios are below the diagonal, p-values are above the diagonal, and the diagonal contains the observed rate of phenotypic evolution. Significant p-values using a conservative Bonferroni correction (α = 0.05/15 = 0.0033) are highlighted in gray along with their corresponding pairwise rate ratios. Observed rates for arboreal, terrestrial, and aquatic species are bolded.

|  | A | C | F | S | T | W |
| --- | --- | --- | --- | --- | --- | --- |
| A | **0.000472** | 0.088 | 0.623 | 0.152 | 0.001 | 0.001 |
| C | 1.630088 | 0.000769 | 0.656 | 0.976 | 0.259 | 0.002 |
| F | 1.262562 | 1.291095 | 0.000596 | 0.678 | 0.260 | 0.010 |
| S | 1.610905 | 1.011908 | 1.275902 | 0.000760 | 0.339 | 0.004 |
| T | 2.211870 | 1.356902 | 1.751890 | 1.373060 | **0.001044** | 0.001 |
| W | 4.737058 | 2.906013 | 3.751941 | 2.940618 | 2.141653 | **0.002236** |


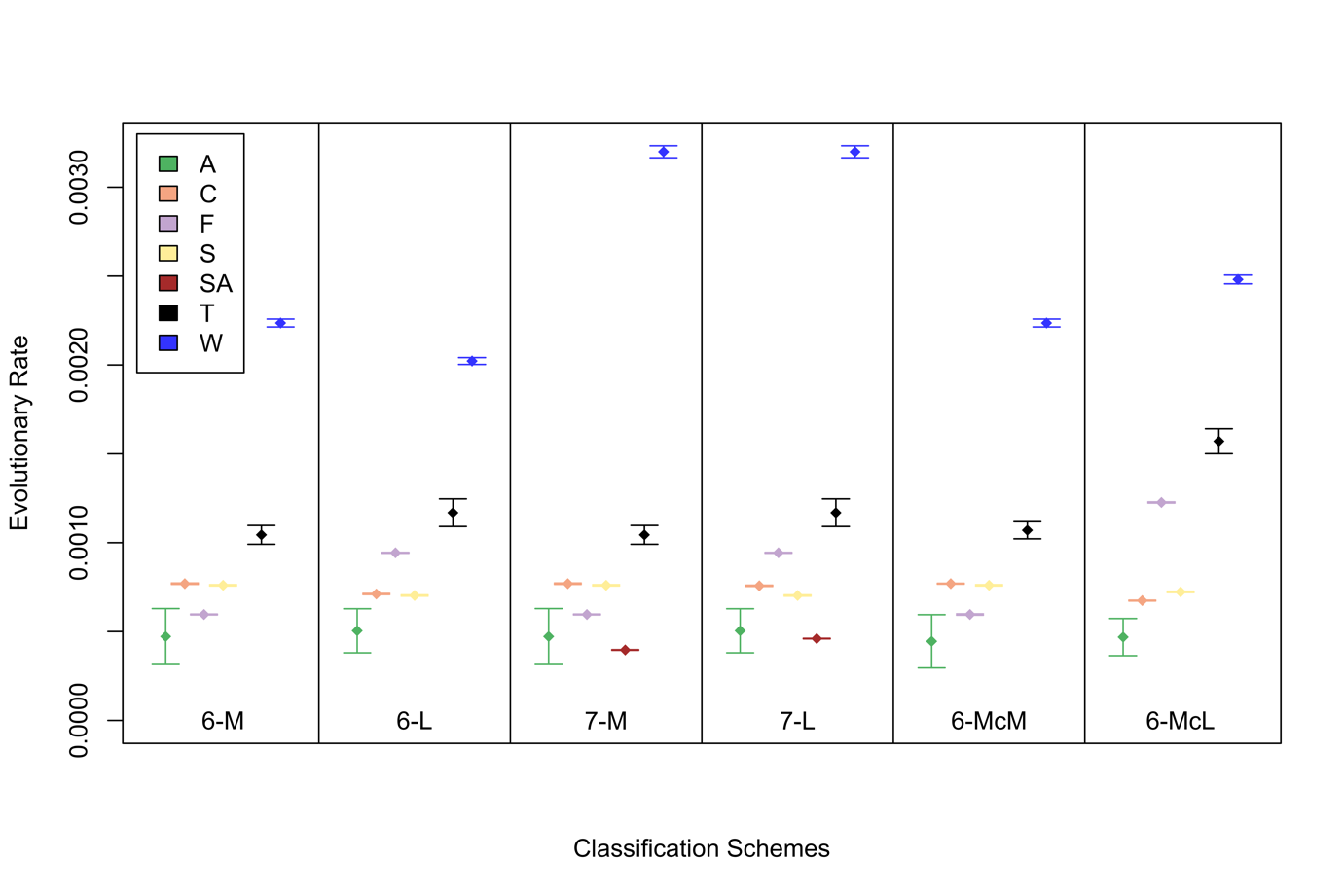


Figure S1. Rates of body shape evolution calculated using different classification schemes. Microhabitat types are arboreal (A), cave (C), fossorial (F), saxicolous (S), semi-aquatic (SA), terrestrial (T), and aquatic (W). Confidence intervals were generated by running analyses on the 1000 posterior chronograms provided by Bonett and Blair (2017). For all classification schemes, arboreal species have a significantly lower rate of phenotypic evolution than terrestrial and aquatic species for at least 94.7% and 98.0% of all posterior chronograms respectively. For classification schemes 6-L, 7-L, and 6-McL, arboreal species also displayed significantly slower rates of phenotypic evolution than fossorial species.

Table S2. Pairwise comparisons of foot shape evolutionary rates across different microhabitat types for 6-M classification scheme on the consensus phylogeny. Microhabitat classifications include arboreal (A), cave (C), fossorial (F), saxicolous (S), terrestrial (T), and aquatic (W). Pairwise rate ratios are below the diagonal, p-values are above the diagonal, and the diagonal contains the observed rate of phenotypic evolution. Significant p-values using a conservative Bonferroni correction (α = 0.05/15 = 0.0033) are highlighted in gray along with their corresponding pairwise rate ratios. Observed rates for arboreal, terrestrial, and aquatic species are bolded.

|  | A | C | F | S | T | W |
| --- | --- | --- | --- | --- | --- | --- |
| A | **0.000005** | 0.102 | 0.817 | 0.484 | 0.001 | 0.001 |
| C | 1.334550 | 0.000007 | 0.327 | 0.655 | 0.076 | 0.001 |
| F | 1.100233 | 1.468316 | 0.000005 | 0.526 | 0.063 | 0.001 |
| S | 1.178442 | 1.132469 | 1.296562 | 0.000006 | 0.034 | 0.001 |
| T | 1.820559 | 1.364175 | 2.003040 | 1.544886 | **0.000010** | 0.001 |
| W | 5.284321 | 3.959628 | 5.813986 | 4.484157 | 2.902582 | **0.000028** |


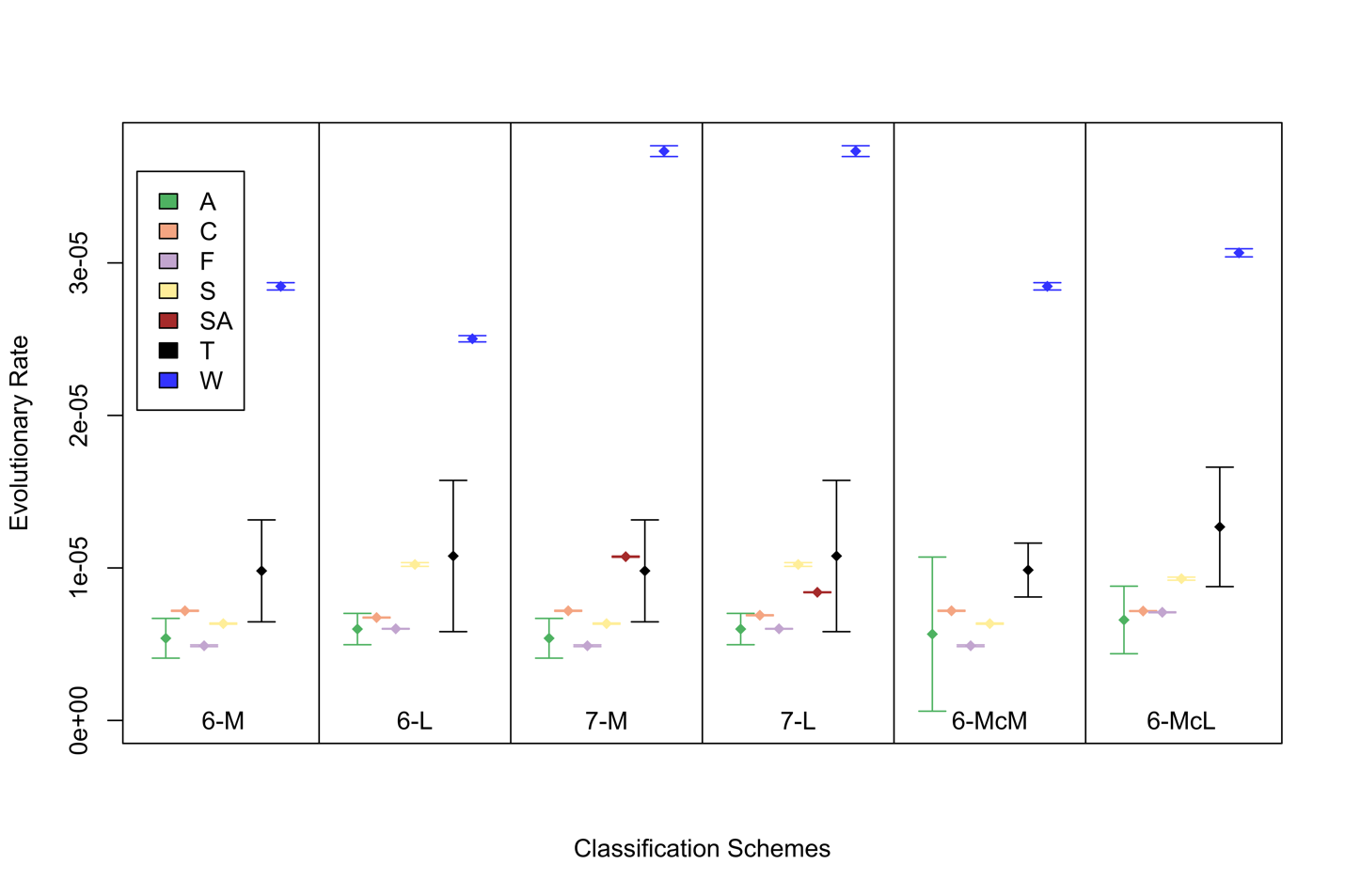


Figure S2. Rates of body shape evolution calculated using different classification schemes. Microhabitat types are arboreal (A), cave (C), fossorial (F), saxicolous (S), semi-aquatic (SA), terrestrial (T), and aquatic (W). Confidence intervals were generated by running analyses on the 1000 posterior chronograms provided by Bonett and Blair (2017). For all classification schemes, arboreal species have a significantly lower rate of phenotypic evolution than terrestrial and aquatic species for at least 91.4% and 98.4% of all posterior chronograms respectively.

Appendix S5. Allometric convergence robustness analyses.

Table S1. Robustness of allometric convergence between arboreal and cave foot shape across classification schemes. All but one scheme show significance at alpha = 0.05, and the one exception is 6-McL with a p value of 0.23. As 6-McL categorizes all species that facultatively use vegetation as arboreal species, we suspect that this list included several species that appear on vegetation anomalously (see McEntire 2016). Thus the foot shape of these species is likely not to have responded to selective pressures associated with climbing.

| **Scheme** | **Distance** | **P-Value** |
| --- | --- | --- |
| 6-M | 0.130 | 0.01 |
| 6-L | 0.102 | 0.02 |
| 7-M | 0.131 | 0.01 |
| 7-L | 0.078 | 0.01 |
| 6-McM | 0.120 | 0.02 |
| 6-McL | 0.012 | 0.23 |


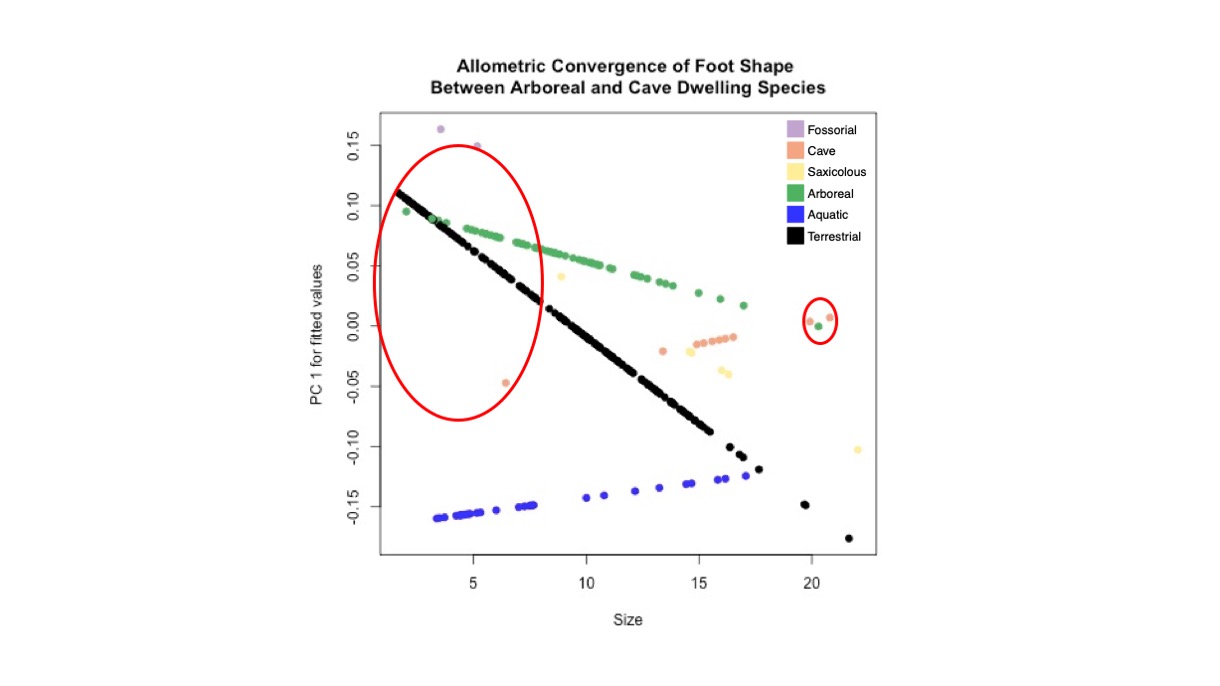


Figure S1. Allometric convergence of arboreal and cave-dwelling foot shape as foot size (centroid size) increases. Circled in red are the predicted foot shapes for small and large foot sizes used to assess significance.
